# Experiences of New Zealand Podiatrists Providing Podiatry Care to People With Foot Osteoarthritis

**DOI:** 10.1002/jfa2.70108

**Published:** 2025-12-17

**Authors:** Prue Molyneux, Mickey Ma, Catherine Bowen, Richard F. Ellis, Keith Rome, Matthew R. Carroll

**Affiliations:** ^1^ School of Allied Health Auckland University of Technology Auckland New Zealand; ^2^ AUT Active Living and Rehabilitation Research Centre School of Allied Health Auckland University of Technology Auckland New Zealand; ^3^ Faculty of Environmental and Life Sciences School of Health Sciences University of Southampton Southampton UK; ^4^ Centre for Sport Exercise and Osteoarthritis Versus Arthritis University of Southampton Southampton UK

**Keywords:** foot osteoarthritis, podiatrists, podiatry care

## Abstract

**Background:**

Current care provided by health professionals for individuals with osteoarthritis (OA) is inconsistent with clinical guideline recommendations. Although OA guidelines have been developed for more commonly studied joints such as the knee and hip, foot OA remains comparatively underrepresented. Despite its high prevalence and significant impact on functional ability, foot OA lacks standardised classification criteria. The absence of clinical guidelines for foot OA underscores its importance as a research priority. Understanding current assessment and management strategies is crucial before designing clinical trials. This study aims to assess New Zealand (NZ) podiatrists' knowledge of foot OA, their assessment practices and their management strategies. Although foot OA is both highly prevalent and functionally disabling, the absence of standardised classification criteria persists.

**Methods:**

A qualitative descriptive approach was employed for data collection and analysis. Using purposive sampling, semistructured interviews were conducted with 10 NZ registered podiatrists. An interview schedule guided discussions on diagnosing and managing foot OA. Interviews were audio‐recorded and transcribed verbatim. Reflexive thematic analysis was used to identify key meanings and patterns within the data.

**Results:**

Five key themes were derived regarding the assessment of foot OA: (1) chief complaint versus incidental finding; (2) obtaining patient history through subjective interviews; (3) targeted objective assessments for foot OA; (4) determining individual biomechanical factors and (5) further investigations. Five themes were identified relating to the management of foot OA: (1) knowledge and language used to provide education about OA; (2) clinical uncertainty necessitates an iterative approach; (3) podiatry administered treatments; (4) referral pathways to other health professionals and (5) management influences.

**Conclusion:**

New Zealand podiatrists utilise a comprehensive diagnostic approach, integrating symptom history, joint mobility assessment and radiographic imaging particularly in the absence of formal diagnostic criteria. Management strategies align with international guidelines, emphasising education, exercise and weight management alongside podiatrist‐led interventions such as foot orthoses and footwear modifications. However, the study highlights several challenges: limited evidence‐based guidance, uncertainty around optimal orthotic strategies and a disconnect between evolving OA knowledge and its application into clinical practice.

## 1 Background

The global impact of osteoarthritis (OA) constitutes a major worldwide challenge for health systems [1]. By 2030, OA is predicted to be the single greatest cause of disability globally with an estimated 35% prevalence [2]. The impact of OA continues to grow due to the ageing population, the rising prevalence of obesity and the lack of definitive treatments to prevent or halt the progression of the disease [3]. Consequently, it is anticipated that there will be an increased demand for health services for the symptoms and disability associated with OA [4]. Current care provided by health professionals for individuals with OA is variable and frequently inconsistent with clinical guideline recommendations [5]. The key components of best evidence for OA care are education and support for self‐management, physical activity, and maintaining healthy bodyweight [6]. Consistent evidence‐practice gaps in OA care are observed in primary care settings globally [7]. Alarmingly, the pass rates for core OA treatments are below 40% in low‐ and middle‐income countries [8]. Building workforce capacity to deliver high‐value care requires a contemporary understanding of OA. Insufficient knowledge by health professionals is a key factor preventing people with OA from receiving appropriate care [7]. Outdated misconceptions of OA persist in clinical practice [9]. Impairment‐based language inadvertently reinforces outdated views of OA and may potentially discourage people from actively participating in their own care [10].

Foot OA results in functional limitations and significant impairments in balance, strength, locomotor ability and negatively impacts work ability [4]. However, the feet are often overlooked as a site of involvement relative to other joints commonly affected by OA [11], even though foot OA prevalence is comparable to more commonly spotlighted areas such as the knee and hip. Although foot OA is both highly prevalent and functionally disabling, the absence of standardised classification criteria persists. This gap is primarily attributed to the condition's heterogeneous clinical presentation, the ambiguity of its signs and symptoms and the reliance on radiographic imaging for definitive diagnosis [12, 13, 14].

The paucity of research on foot OA presents a significant challenge for clinicians, limiting evidence‐based decision‐making and complicating the evaluation of treatment effectiveness. Several nonsurgical interventions have been trialed for foot OA with inconclusive results, including shoe stiffening inserts [15, 16, 17], foot orthoses [18, 19, 20], intra‐articular corticosteroid injection [21, 22], intra‐articular hyaluronan [23], rocker‐sole footwear [24] and general analgesic advice [4, 25]. However, the lack of randomised controlled trials (RCTs) evaluating these interventions remains a major limitation. Furthermore, there is a notable absence of consensus on podiatric assessment and management strategies for foot OA with current practices often guided by clinician experience rather than robust clinical guidelines. The adoption of new interventions and technologies in routine practice also appears limited with few studies reporting on implementation outcomes or knowledge mobilisation strategies. As a result, clinicians are frequently left to interpret and apply emerging evidence independently, contributing to variability in care and uncertainty in clinical decision‐making.

The absence of clinical guidelines for foot OA underscores its importance as a research priority. Foundational research is essential to establish diagnostic criteria for foot OA and determine what constitutes usual care in general practice [26]. Understanding current assessment and management strategies is essential before designing clinical trials and developing evidence‐based recommendations. In the United Kingdom (UK), individuals with foot OA typically present to general practitioners or podiatrists as their initial point of care [27]. In Australia, general practitioners predominantly adopt pharmacological approaches for managing foot OA [25]. Conversely, podiatrists in both Australia and the UK report utilising a combination of therapeutic strategies, including footwear modifications, orthotic devices and pharmacological treatment [28]. However, there is currently no available evidence on how New Zealand (NZ) podiatrists assess and manage foot OA. Therefore, this study aimed to investigate how podiatrists in NZ currently assess and manage foot OA. The findings are expected to contribute knowledge of podiatric practice in NZ, inform clinical decision‐making and shape future research directions in this area.

## 2 Methods

### 2.1 Positionality Statement

This research was conducted by a multidisciplinary team of podiatrists and allied health academics based primarily in New Zealand with additional expertise from the United Kingdom. The lead researchers are experienced clinicians and academics with extensive backgrounds in musculoskeletal podiatry, qualitative research and allied health education. Their professional roles and clinical experience have shaped the study's focus on real‐world assessment and management practices for foot OA in NZ.

The research team acknowledges that their clinical backgrounds and ongoing engagement with podiatric practice may influence their interpretation of participants' experiences and the thematic analysis. The study's qualitative descriptive methodology, grounded in naturalistic inquiry, was chosen to centre the voices of practising podiatrists and to reflect the realities of clinical care. Reflexivity was maintained throughout the research process, including the use of reflexive journals, independent coding, and regular team discussions to challenge assumptions and minimise bias.

The researchers recognise that their positions within academic and clinical networks may have facilitated recruitment and shaped the dialogue during interviews. Efforts were made to ensure a diverse sample and to promote trustworthiness through de‐identification, participant transcript review, and adherence to established qualitative research standards. The team is committed to advancing evidence‐based, patient‐centred care for people with foot OA and acknowledges the importance of transparency regarding their own perspectives and potential influences on the research process.

### 2.2 Design

This interpretivist study used a qualitative descriptive methodology [29], which enabled a meaningful understanding of participants' experiences and perceptions of diagnosing and managing foot OA. This drew on the principles of naturalistic inquiry, which requires analysis of the data to be reflected in the descriptions given by participants and the findings to be reported using participants' own words and quotes [30]. An inductive approach to analysis allowed the data to determine the themes. The reporting of the study is in accordance with the Consolidated Criteria for Reporting Qualitative Research (COREQ) checklist [31] (Supporting Information S1: Appendix S1).

### 2.3 Participants

Participants were purposively sampled through professional networks and social media (X and Facebook) between December 2022 and February 2023. Participants were included if they were registered podiatrists in NZ, had at least five years of clinical experience and had a predominantly (∼60%) biomechanical clinical caseload. These criteria were chosen to ensure the targeted recruitment of participants with sufficient experience in the assessment and management of musculoskeletal‐based pathology, thereby generating high‐quality dialogue that specifically addressed the research aim. Recruitment efforts were made to ensure a diverse sample from a broad range of NZ regions. Ethical approval was obtained from the Auckland University of Technology Ethics Committee (22/301), and all participants provided oral or written informed consent prior to data collection.

### 2.4 Data Collection

Semistructured interviews were conducted to explore the participants' individual perspectives. The research team developed an interview schedule (Supporting Information S2: Appendix S2) to guide discussions on diagnosing and managing foot OA. The interview process was initially piloted with an experienced qualitative researcher, who was independent of the research team. Following this, the interview schedule was refined and subsequently piloted with an experienced podiatrist. All interviews were conducted by the first author (PM, PhD), an academic and practising podiatrist. Open‐ended questions were used to mitigate potential response bias. Prompts encouraged focused discussion, with regular summaries shared during interviews and participants were assured their experiences were valuable. Participants attended a single interview either in person at the Auckland University of Technology or online via Zoom. Interviews were audio recorded, transcribed using Otter.ai (Otter version 3.66.250106.1008) and reviewed for accuracy. Transcriptions were anonymised, and participants were invited to review their individual transcription for completeness. Interview duration ranged from 43 to 61 min.

As a means of enhancing the rigour of the qualitative data collection, analysis, and interpretation processes, a range of strategies were employed to promote credibility, transferability, dependability and confirmability. These included adherence to the semistructured interview guide, audio recording interviews, transcribing verbatim and cross checking within the research team [32, 33]. All data were de‐identified to promote trustworthiness of the data analysis process.

### 2.5 Data Analysis

Data collection and analysis occurred simultaneously and iteratively, this created new insights and questions were rephrased as needed, which influenced subsequent interviews and analyses. Interviews continued until the authors considered that sufficient information power [34] was achieved by the clear research aim, diversity of participants and the depth of discussions during interviews. To understand the podiatrists experience of managing foot OA, data analysis was performed using a reflexive thematic analysis approach [35, 36]. Using a reflexive journal, PM and MM maintained reflexivity by reading and rereading the transcripts as well as making notes about participants' remarks and how those remarks were understood and interpreted. Reflective thematic analysis allows the generation of new meaning by producing patterns (themes) within the data. This included data familiarisation by reading the transcripts and listening to the audio recordings multiple times to immerse the researcher in the data [37]. Transcripts were then manually coded by PM and MM with initial codes and concepts reviewed by a third author (MC). Coding was undertaken separately for the assessment and management of foot OA. Multiple iterations of coding were undertaken as part of a recursive approach to the analysis and were discussed with other members of the research team. The generated codes were then grouped into potential themes and subthemes. These were then reviewed to determine a clear distinction between each theme. The final themes were defined, named and agreed upon by all authors. Supporting Information S3: Appendix S3 provides an example of an audit trail. Illustrative quotes from transcripts were selected to provide evidence of each theme.

## 3 Results

Ten NZ‐registered podiatrists, all practising in the private health sector, participated in the study. The participant characteristics are presented in Table 1.

**TABLE 1:** Participant demographics.

ID	Sex	Region of NZ	Years of practice	Ratio of MSK	Frequency of treating patients with foot OA
P1	Male	Auckland	11–15 years	100% MSK	At least 1 per month
P2	Male	Auckland	11–15 years	60% MSK	At least 1 per week
P3	Male	Wairarapa	Over 20 years	70% MSK	Daily
P4	Female	Auckland	16–20 years	60% MSK	At least 1 per week
P5	Female	Christchurch	11–15 years	70% MSK	At least 1 per week
P6	Female	Christchurch	6–10 years	80% MSK	Daily
P7	Female	Auckland	11–15 years	60% MSK	At least 1 per week
P8	Female	Auckland	16–20 years	70% MSK	Daily
P9	Female	Waikato	11–15 years	60% MSK	Daily
P10	Male	Christchurch	Over 20 years	80% MSK	Daily

### 3.1 Assessment of Foot OA

**FIGURE 1:**
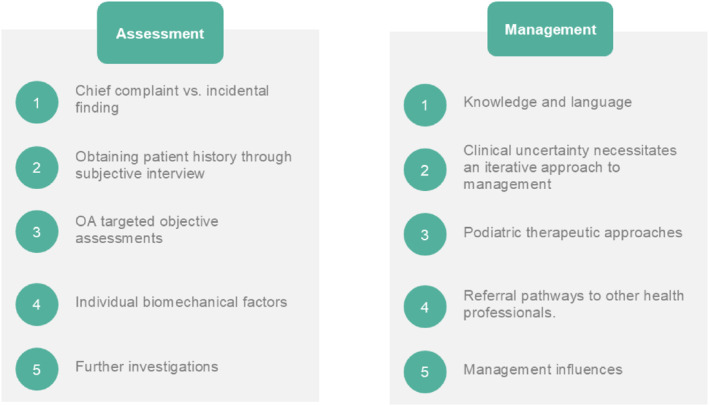
Themes regarding the assessment and management of foot OA.

Five key themes were derived regarding the assessment of foot OA (Figure 1): (1) chief complaint versus incidental finding; (2) obtaining patient history through subjective interviews; (3) targeted objective assessments for foot OA; (4) determining individual biomechanical factors and (5) further investigations.

#### 3.1.1 Theme 1: Chief Complaint Versus Incidental Finding

Chief complaint versus incidental finding was a recurring theme, reported by eight participants (P1, P2, P3, P4, P5, P8, P9, P10). Participants often reported that people with suspected or previously diagnosed foot OA rarely report it as their chief complaint. Foot OA was usually identified as a secondary finding during consultations:

In my experience, OA is more commonly presented as a secondary finding rather than the patient’s main concern. When I asked patients about it, they have often already spoken with their GP and believe nothing can really be done.

(P8)

Whether foot OA was the primary or secondary patient concern, participants emphasised the importance of identifying OA due to its impact on locomotor ability and potential risk for the development of other lower limb pathologies:

I will flag first MTP joint OA as a risk factor for the development of other lower limb conditions. For example, plantar heel pain or Achilles tendinopathy. This could be because of the limited range of motion that is often associated with OA, which can then result in greater strain on other structures.

(P3)

Some patients come in knowing they have foot osteoarthritis, which has been diagnosed by imaging, but they are unaware of the impact that joint has on the rest of the foot.

(P8)

#### 3.1.2 Theme 2: Obtaining Patient History Through Subjective Interview

Obtaining patient history through subjective interview was a recurring theme, reported by all participants (P1, P2, P3, P4, P5, P6, P7, P8, P9, P10). The complexity of diagnosing foot OA is reliant on obtaining key information about patient‐reported symptoms, specifically the presence and pattern of pain and stiffness:

I would ask questions related to location of pain, severity of pain, which movements exacerbated pain … do they get pain with certain footwear or with end‐range of motion.

(P8)

The pattern might be stiffness, following a period of rest, with a reduction in pain as the joint warms up or movement takes place and then an increase in pain as the joint becomes fatigued.

(P3)

Most participants considered external factors to assess the risk of developing foot OA. Many participants noted a link between foot OA and sports involving intense impact and torsional loading:

If someone’s played a lot of football, or a sport where there’s repetitive loads to that joint, I might suspect OA a lot sooner than in someone who has lived a fairly sedentary lifestyle.

(P1)

#### 3.1.3 Theme 3: Foot OA Targeted Objective Assessments

Foot OA targeted objective assessments was a recurring theme, reported by all participants (P1, P2, P3, P4, P5, P6, P7, P8, P9, P10). Most participants reported using a standardised performance or impairment measure as part of their diagnostic methods. The most common were the first MTPJ range of motion, direct palpation, lunge test, visual gait assessment, Foot Posture Index, and presence of crepitus:

Pain on the dorsal aspect of the joint, combined with restriction of first MTP joint dorsiflexion, leads to the belief that there may be some dorsal lipping or change to articular surfaces that may restrict movement.

(P1)

Some participants acknowledged that OA is not a one‐stage disease and mentioned utilising objective assessments to evaluate the severity of OA:

I would consider pain levels and consistency of pain levels, along with severe joint stiffness. If I really needed to be sure how bad the joint was, I would refer for an X‐ray to really see and assess the joint damage.

(P9)

#### 3.1.4 Theme 4: Individual Biomechanical Factors

Individual biomechanical factors was a recurring theme, reported by seven participants (P1, P2, P3, P4, P6, P8, P10). Additional assessments aimed at determining individual contributing or compensatory biomechanical factors were also described:

I would assess to see if there are any modifiable biomechanical factors that could be addressed to reduce load on the joint, things like weight … ankle restriction.

(P8)

Additionally, participants sought to improve understanding of the patients walking or running kinematics and the impact foot OA may have on individual gait patterns:

If someone presented with first MTP joint OA, I would perform a gait assessment to assess their loading during the propulsion phase of gait … for midfoot OA, I would be more interested in assessing their foot during loading and midstance.

(P4)

#### 3.1.5 Theme 5: Further Investigations

Further investigations was a recurring theme, reported by nine participants (P1, P2, P3, P4, P5, P6, P7, P9, P10). Direct referrals for imaging investigations were commonly used to exclude alternative differential diagnoses and confirm the diagnosis of foot OA. Plain radiography was the most frequently utilised imaging modality. However, the timeframe for radiographic investigations varied among podiatrists. With many participants only referring for imaging after exhausting conservative treatment modalities, with limited success. Opposed to utilising imaging as a means of confirming diagnosis from the outset:

There’re probably two reasons why I would refer for X‐ray. Firstly, if that patient who presented to me is seeing multiple other providers and is not getting any results … Otherwise, if first presented with foot pain, what is radiology going to change in my treatment.

(P10)

New Zealand podiatrist can refer for ultrasound imaging. However, this imaging modality was rarely utilised. There appeared to be some uncertainty with its clinical utility:

I don’t believe ultrasound will show me bony damage, you know … I am unsure of the value of ultrasound for OA.

(P1)

I have become more aware of the capacity of ultrasound imaging in recent years … but I’m still unsure if it will give me the information I need.

(P2)

Some participants reported they referred for imaging to assess the severity of foot OA, subsequently informing their management approaches:

X‐ray to tell me how aggressive I can be with treatment and whether I can restore movement to the joint or whether I have to restrict joint movement.

(P1)

The OA phenotype influenced whether imaging was used to make a diagnosis or inform their management approach. Uncertainty was expressed when establishing a diagnosis for midfoot OA based on clinical assessments alone. Whereas for first MTPJ OA, imaging was often employed to inform management approaches:

For midfoot OA, I would refer for imaging to confirm diagnosis, whereas for first MTP joint, I am using imaging to see how aggressive I can be with treatment.

(P1)

### 3.2 Management of Foot OA

Five themes were identified relating to the management of foot OA (Figure 1): (1) knowledge and language; (2) clinical uncertainty necessitates an iterative approach to management; (3) podiatry administered treatments; (4) referral pathways to other health professionals and (5) management influences.

#### 3.2.1 Theme 1: Knowledge and Language

Knowledge and language were a recurring themes reported by nine participants (P1, P2, P3, P4, P5, P6, P8, P9, P10). Improving patient understanding through clear education and communication was viewed as a key concept to an effective management programme. When asked to define OA, emphasis was placed on the structural deterioration of a joint surface. Terms such as ‘wear and tear’, ‘bone on bone’ and degenerative were the most commonly reported:

‘Wear and tear arthritis’ and ‘joint space narrowing’ are the simple words that I would use to explain OA to patients.

(P2)

‘Bone on bone’ would be the words I would use to describe OA to a patient.

(P3)

One participant mentioned the inflammatory component of OA:

I would discuss breakdown of cartilage, osteophytes, inflammation and biomechanical load.

(P3)

Participants reinforced checking in with the patient's understanding of OA, signposting that OA is often poorly understood:

In my experience, people go one of two ways. They either go, ‘I’m getting old. It’s fine. It’s part of it’, or they kind of catastrophise the word ‘arthritis’. I think both of those groups need some kind of improved awareness to actually what it is and to a certain extent to a normal joint that’s lived a life but it’s not the end of the world. We can manage it. We can still live a healthy, happy life with foot osteoarthritis so long as you’re managing properly.

(P1)

Patient education centred on reducing pain and improving patient function. This required debunking many misconceptions about what can be done for foot OA:

Patients often present with the belief that nothing can really be done or even report they’ve been told nothing can be done by another healthcare professional.

(P9)

For many, education also encompasses load management principles involving activity modification to ensure physical activity is achievable and maintained:

I will always stress the importance of maintaining activity levels and reinforce that it’s not going to make their osteoarthritis worse, which is often the patient's fear.

(P9)

The importance of a clear management plan, which is patient‐centred, was highlighted:

You might have someone that’s 60 plus that comes in that um … you know, wants to walk but can't walk because of the pain. But you also in the next week, you might have a 32‐year‐old that comes in that wants to do a marathon but is experiencing pain in their big toe joint as well. So, my management is targeted to the individual, their lifestyle and their goals.

(P9)

A key concept of patient education addressed modifiable risk factors such as body mass and physical activity. This aspect of education was focused on the fact that excess weight will produce increased load on the joint. The metabolic contribution to OA was not discussed:

I often find weight management and education go hand in hand with encouraging patients to keep active, there is often a fear that they will worsen their OA. My focus is to just get them walking, swimming or cycling combined with a strengthening programme.

(P3)

Most acknowledged the vicious cycle that many people living with OA find themselves in. Where they're aware they need to exercise to lose weight but are in too much pain to do so:

I understand that they're in pain. I also understand because they're in pain, they're not able to exercise as well as they could. So, I empathise with patients, and I often say, my job is to try and control the pain so you can exercise.

(P3)

#### 3.2.2 Theme 2: Clinical Uncertainty Necessitates an Iterative Approach to Management

Clinical uncertainty necessitates an iterative approach to management was a recurring theme reported by six participants (P1, P4, P7, P8, P9, P10). Due to the ambiguity about how some patients may respond, participants often reported clinical uncertainty when determining their management approach. Particularly with regards to clinical reasoning for selected modifications and materials as part of their orthotic prescription:

Different treatments work for different people, so managing foot OA often involves a trial‐and‐error approach. The effectiveness of a treatment depends on the specific joint affected and individual patient factors.

(P8)

A recurring uncertainty in clinical decision‐making was knowing when to enhance or restrict joint motion and at what stage of the disease process. The consensus among participants was to facilitate movement until it is too painful to do so:

If a patient has first MTP joint OA, it is not always clear if I should be trying to facilitate joint movement with a 2‐to‐5 pad or I should be trying to unload the joint with a Morton’s extension … I will always aim to facilitate movement first before trying to restrict it.

(P8)

#### 3.2.3 Theme 3: Podiatric Therapeutic Approaches

Podiatric therapeutic approaches was a recurring theme reported by all participants (P1, P2, P3, P4, P5, P6, P7, P8, P9, P10). Participants employed a range of treatment strategies. However, no single effective approach to managing foot OA was described. The interventions selected were dependent upon the stage of disease and individual patient factors.

##### 3.2.3.1 Footwear

Footwear was considered the most important management approach. The most common recommendation was rocker‐sole shoes to facilitate sagittal plane motion and reduce joint loading particularly for the management of first MTPJ OA:

Education on footwear is fundamental; we don’t do anything until we have the footwear right.

(P3)

Footwear recommendations for midfoot OA were focused on stability, reducing dorsal compression around the joint, accommodating for bony prominences, lacing techniques and arch support:

I would typically recommend stability over cushioning for midfoot OA. Once they have the strong base of support, I can then add to it with arch pads or orthoses.

(P2)

Additional considerations when assessing footwear included ensuring sufficient toe‐box room and evaluating seams that run across the inside of the shoe, which may cause potential irritation.

##### 3.2.3.2 Strapping

Strapping was applied to guide clinical decision‐making to determine the potential effectiveness of orthotic therapy by changing the position or resisting movement through a joint:

I will often apply a strapping technique to immobilise and resist dorsiflexion of the first MTPJ during the propulsive phase of gait … this will help determine the effectiveness of orthoses for first MTP joint OA.

(P8)

Participants also reported using strapping to reinforce the possibility of managing OA symptoms, facilitating trust in the patient‐physician relationship:

Patients often present believing nothing can be done for their OA. Strapping is a useful tool to prove to the patient that their symptoms can be reduced.

(P9)

##### 3.2.3.3 Foot Orthoses

All participants indicated that they would prescribe foot orthoses to manage foot OA, irrespective of the joint affected. The common objective was to alter the magnitude and location of forces along with addressing other biomechanical factors. The chronic nature of OA often justified the selection of custom‐made over prefabricated foot orthoses. With custom devices providing greater durability and wider flexibility in the prescription of the device:

I tend to gravitate towards customs over prefabs for chronic conditions such as OA. It’s not going to resolve, so customs are going to last longer and allows me to be more flexible in my prescription.

(P3)

Some participants start with prefabricated foot orthoses to prove efficacy as part of the short‐term management before progressing to a customised device in the long‐term:

I find prefabs gain confidence with the patient and allows us to modify and change really easily, then commonly progress to a more customised device in 1 to 2 years.

(P10)

###### 3.2.3.3.1 Orthotic Modifications

Most participants recognised that OA is a progressive, multistage disease, and the severity of an individual's foot OA influenced their orthotic prescription.

####### 3.2.3.3.1.1 First MTPJ OA

A consistent finding was the uncertainty around targeting the appropriate modification for the stage of disease. Specifically, it is important to recognise at what point the objective of the orthotic changes from facilitating movement (i.e., with a kinetic wedge modification [38]) to restricting movement (i.e., Morton's extension modification [39]). For the MTPJ OA, a Morton's extension was the preferred modification to reduce load on the joint. The caveat is that a Morton's extension was only considered once it was established that enhancing joint movement was no longer feasible:

The hardest factor to determine is at what point do we switch from trying to facilitate movement to restrict movement. For example, moving from a kinetic wedge to a Morton’s extension.

(P5)

The density of the material used for Morton's extension varied and often depended on patient acceptability and comfort. Materials ranged from soft foam (e.g., Poron) to a hard, in‐built shell extension:

I have found patients don’t necessarily tolerate the in‐shell Morton’s extension. I also think it's dependent on how progressed they are. So, I will start conservative with a Poron or an EVA extension instead.

(P9)

In some instances, carbon fibre plates were used when a greater degree of immobilisation was required. However, there was an inconsistency in its application. Some participants recommended a shoe with an inbuilt carbon plate to facilitate propulsion. Others had administered a full‐length carbon fibre plate that was inserted underneath the shoe sock liner, or some even combined a carbon plate with an orthotic device. Carbon plates were used with caution due to patient acceptability and comfort:

I've used an in‐shell Morton's extension, but I actually found that most people didn't tolerate it too well because it dorsiflexed or elevated the first MPJ too much in the shoe, that it added too much pressure onto what was already a tender joint. So, in those cases, I actually found just using the carbon plates actually worked better.

(P4)

####### 3.2.3.3.1.2 Midfoot OA

For midfoot OA orthotic prescription, arch support was the key objective:

The focus for midfoot OA is to try and provide a dorsal moment. You would keep that arch contour quite high with an arch fill pad.

(P9)

##### 3.2.3.4 Exercise Prescription

In addition to footwear education and orthotic prescription, the inclusion of exercises was often recommended in conjunction:

I will often incorporate manual therapy, joint mobilisation, stretching. The aim of this is to try and reduce proximal compensations, tightness, for example ankle equinus.

(P3)

#### 3.2.4 Theme 4: Referral Pathways to Other Health Professionals

Referral pathways to other health professionals was a recurring theme reported by eight participants (P1, P2, P4, P6, P7, P8, P9, P10). Referral pathways to wider interdisciplinary team members for further investigation or management were reported. Referral practices were considered for patients who experienced limited improvement with conservative podiatric treatment.

As it stands, NZ podiatrists have recently been granted prescribing rights, but there are currently no designated prescribers. Some participants discussed pharmacological management with their patients. This involved recommending over‐the‐counter medications or referral to a pharmacist or General Practitioner (GP):

So pharmaceutically, the prophylactic use of nonsteroidals … but I would recommend consulting a pharmacist or GP.

(P3)

More invasive therapies performed by an orthopaedic or specialist were only considered as a last resort after exhausting conservative modalities with limited success:

It must be pretty progressed for a surgical referral. If we were struggling to get it under control conservatively, I would raise the fact that it is an option. But my view is the patient has to reach a point where symptomatically they’re not coping.

(P1)

#### 3.2.5 Theme 5: Management Influences

Referral pathways to other health professionals was a recurring theme reported by five participants (P1, P2, P6, P8, P9). Participants reported their management approaches have evolved over their career and are influenced by clinical experience, previous patient outcomes and advancements in knowledge. Individual patient goals were also reported to influence management approaches:

How active do they want to be? … Some patients come in and just want to do their day‐to‐day activity without pain, and that's quite a different consideration to if they're still wanting to go hiking and running.

(P1)

Cost was reported as a barrier and often influenced management approaches. Specifically, when it came to prescribing foot orthoses and/or referrals for new footwear and imaging that lead to additional patient costs:

A lot of my patients don’t have the privilege of purchasing new shoes and orthoses. For that reason, my management approach may not always be best practice but it’s about making the most from limited resources.

(P6)

Health inequities in NZ were highlighted as a substantial influence. Specifically, regarding access within the regions of NZ:

Access and cost are huge barriers in the regions. For that reason, unfortunately my management approach is driven by cost not necessarily proven efficacy.

(P9)

## 4 Discussion

This study is the first to explore the assessment and management practices employed by podiatrists in NZ for patients with foot OA. Participants described a thorough approach to evaluation, emphasising symptom history, joint range of motion and plain radiography to support diagnosis and assess disease severity. This method enabled the identification of individual biomechanical factors essential for developing personalised and targeted management plans. The reported management strategies align with the National Institute for Health and Care Excellence guidelines for peripheral joint OA, including education, exercise and weight loss when appropriate [40]. These guidelines are supported by the Osteoarthritis Research Society International [41], the Royal Australian College of General Practitioners [42] and The European League Against Rheumatism (EULAR) [43]. Key management strategies included patient education and podiatrist‐led interventions, such as the prescription of foot orthoses. These treatments were often guided by traditional perspectives on OA with some uncertainty regarding the optimal timing and patient selection for specific modifications.

In the absence of clinical guidelines or diagnostic criteria for the diagnosis of foot OA, NZ podiatrists rely on their clinical experience and knowledge using methods comparable to practices in Australia and the UK [44]. Despite no acknowledgement of evidence‐based practice, clinical observations and assessment techniques for first MTPJ OA aligned with the diagnostic rule proposed by Zammit and colleagues [45] and recommended clinical assessments to inform management proposed by Paterson and colleagues [46]. The clinical diagnosis of midfoot OA poses challenges in isolating and assessing tenderness, stiffness and deformity due to the proximity of the midfoot joints. Physical examinations may have limited utility in distinguishing the presence or absence of symptomatic midfoot OA [47]. Consequently, midfoot OA requires imaging to augment clinical history and examination. Plain radiography was the most utilised imaging modality reported to confirm diagnosis and/or determine the severity of foot OA similar to Australian data [4, 48] and a bibliometric analysis of foot OA research [49]. This high usage is likely due to insufficient clinical diagnostic criteria for foot OA. The EULAR recommendations for peripheral joint OA highlight the need for more research on the impact of imaging on foot OA management [50].

A lack of clinician knowledge remains a significant barrier to appropriate care for individuals with OA globally [7]. Education was identified by NZ podiatrist as a critical component of effective foot OA management, highlighting the need for a contemporary understanding of the disease. However, participants often overlooked the complex, multitissue nature of OA, instead on structural changes alone. The study revealed a disconnect between advances in OA knowledge and their application into clinical practice particularly evident in the language used to educate patients. Clinicians' terminology can negatively influence patients' beliefs about OA management [5]. Podiatrists observed that many patients perceive OA as an inevitable consequence of ageing, which fosters misconceptions about available treatment options. Despite efforts to challenge these beliefs, podiatrist frequently relied on impairment‐based language, inadvertently reinforcing outdated views of OA as a degenerative condition. Phrases such as ‘wear and tear’ and ‘bone on bone’ are now recognised as obsolete, inaccurate and potentially harmful [9, 51, 52, 53]. Such language affirms patients' perceptions by reinforcing the misconception that joints have a finite lifespan and that physical activity exacerbates damage, ideas that contradict current management strategies focused on pain relief and functional improvement. Despite evolving scientific understanding, OA continues to suffer from an identity crisis, shaped by a deeply entrenched disease and impairment discourse [10, 54]. This underscores the urgent need to shift the OA narrative in NZ. Healthcare providers should adopt a health promotion approach that emphasises modifiable factors and empowers individuals to manage symptoms effectively [55]. As the field of foot OA evolves, researchers must ensure that emerging knowledge is mobilised and integrated into clinical practice.

Prescribing foot orthoses and appropriate footwear were central to managing foot OA. These podiatry‐led interventions help redistribute load, cushion, restrict, immobilise or facilitate joint motion. Despite the high prevalence of foot OA, few RCTs have investigated management strategies for foot OA. Orthoses, shoe‐stiffening inserts, rocker‐sole footwear, and intra‐articular hyaluronan have been trialled for first MTPJ OA with inconclusive results [17, 18, 23, 24, 56]. Rocker‐sole shoes were highly recommended for first MTPJ OA to reduce dorsiflexion needs and peak pressure under the first MTPJ [24]. Menz (2016) demonstrated that prefabricated foot orthoses and rocker‐sole insoles reduced first MTPJ OA symptoms [24]. Paterson (2022) found contoured foot orthoses no more effective than flat sham insoles for first MTPJ OA [18]. Carbon fibre inserts were recommended by some participants; however, these were frequently employed in combination with foot orthoses. This approach differed from the trial protocol, which excluded individuals unwilling to discontinue the use of any foot orthotic devices [17]. Notably, carbon‐fibre shoe‐stiffening inserts have been shown to improve foot pain and function in one in four participants with first MTPJ OA [17]. For midfoot OA, shoe‐stiffening inserts and foot orthoses were recommended. Both interventions have been shown to improve pain and function in midfoot arthritis [19, 20]. However, the long‐term effects of these interventions are unknown [57]. The paucity of research makes it difficult for clinicians to provide evidence‐based practice and determine the efficacy of interventions.

Orthotic modifications were guided by the patient's disease stage and individual biomechanical characteristics. However, there was notable uncertainty regarding whether the primary goal of orthotic prescription should be to restrict or facilitate joint movement. As a result, clinicians often relied on an iterative approach to management. This reflects the understanding that OA is a progressive condition rather than a single‐stage disease, and a patient's position along the disease continuum (e.g., early vs. established) may determine treatment efficacy. Despite this, interventions have primarily been investigated in populations with radiographically confirmed foot OA typically representing more advanced stages of the disease. Radiographic confirmation identifies individuals who already have more established structural joint damage and/or functional impairment often beyond the optimal window for disease‐modifying interventions. This focus on established or end‐stage OA may partly explain the limited effectiveness of current conservative treatments. This underscores the disconnect between clinical decision‐making and the inclusion criteria of RCTs. Consequently, podiatrists face uncertainty in predicting, which patients are likely to benefit from conservative care. This necessitates future research to identify a homogeneous subset of people who present with symptoms prior to significant radiographic changes. Targeting this earlier stage of disease may offer a greater opportunity for conservative interventions to alter disease progression and symptomatic consequence.

This study should be considered in light of some limitations. Firstly, some participants were known to the interviewer, which may have introduced bias during data collection. However, all interviews were analysed independently by a second member of the research team who was unknown to all participants. Secondly, interviews were facilitated by an experienced podiatrist, who also contributed to the analysis and interpretation of the results, which may have introduced personal bias. However, reflective thematic analysis recognises that research occurs due to the subjectivity of the researcher and does not attempt to eliminate it [37]. In addition, the podiatric experience of the interviewer ensured that the discussions were focused and specific to the research aim. Finally, it is important to highlight that this study is an interview‐based investigation involving healthcare professionals (specifically, podiatrists) not an evidence‐based clinical guideline. The findings reflect the current practices of NZ podiatrists who reported managing individuals with foot OA. Rather than providing guidance on best practice, this study offers insights into common approaches to the assessment and nonsurgical management of foot OA.

## 5 Conclusion

New Zealand podiatrists utilise a comprehensive diagnostic approach, integrating symptom history, joint mobility assessment and radiographic imaging particularly in the absence of formal diagnostic criteria. Management strategies align with international guidelines, emphasising education, exercise and weight management alongside podiatrist‐led interventions such as foot orthoses and footwear modifications. However, the study highlights several challenges, including limited evidence‐based guidance, ambiguity surrounding optimal orthotic strategies, and a disconnect between evolving OA knowledge and its translation into clinical practice. These challenges not only hinder effective care but also highlight an urgent need for systemic change. To address this, clinical trial designs must evolve, prioritising the development of validated diagnostic tools and targeted interventions. Equally important is the creation and dissemination of up‐to‐date, accessible educational resources that empower clinicians to apply the latest evidence with confidence. Additionally, researchers must embed implementation strategies early in the design phase of randomised controlled trials to ensure that new evidence translates effectively into practice.

## Author Contributions

**Prue Molyneux:** conceptualisation (lead), formal analysis (lead), funding acquisition (lead), investigation (lead), methodology, writing – original draft (lead), writing – review and editing. **Mickey Ma:** formal analysis, writing – review and editing (equal). **Catherine Bowen:** conceptualisation, funding acquisition, methodology, supervision (equal), writing – review and editing (equal). **Richard F. Ellis:** conceptualisation, funding acquisition, methodology, supervision (equal), writing – review and editing (equal). **Keith Rome:** conceptualisation, funding acquisition, methodology, supervision (equal), writing – review and editing (equal). **Matthew R. Carroll:** conceptualisation, funding acquisition, methodology, supervision (lead), writing – original draft, writing – review and editing (equal).

## Funding

This study was funded by the Arthritis New Zealand. This organisation had no role in the study design, collection, analysis, interpretation of the data or in the decision to submit the article for publication.

## Conflicts of Interest

Prue Molyneux is on the editorial board for the Journal of Foot and Ankle Research. Mickey Ma, Catherine Bowen, Richard F. Ellis, Keith Rome and Matthew R. Carroll declare no conflicts of interest in relation to this work.

## Supporting information


Supporting Information S1

Supporting Information S2

Supporting Information S3

## Data Availability

The data that support the findings of this study are available from the corresponding author upon reasonable request.
