# Supplementary material for: Experiences of New Zealand Podiatrists Providing Podiatry Care to People With Foot Osteoarthritis
Source: J Foot Ankle Res. 2025 Dec 17;18(4):e70108. doi: 10.1002/jfa2.70108 (PMC12712349; doi:10.1002/jfa2.70108)
Supplement: Supplementary file 2 — Supporting Information S2 [file JFA2-18-e70108-s001.docx]

**INTERVIEW QUESTION SHEDULE**

**Investigating the experience of podiatrists providing podiatry care to people with foot osteoarthritis**

Kia Ora

Firstly, I’d like to thank you for your time and your interest in our research. I’m PM I’m a registered podiatrist, researcher, and lecturer at AUT. The goal of this interview is to share your thoughts and experiences around the assessment and management of foot osteoarthritis. Just to remind you – our discussion today will be recorded, but all the data will be de-identified to ensure that you are not identified personally in any report/publication arising from the research.

Before we start, I would like to mention

- There are no right or wrong answers. I encourage you to speak freely today during our discussions.
- Your individual experience and opinions are valuable.
- This interview should take around 45 to 60 minutes, you don’t have to contribute to a specific question if you don’t want to, and you are free to end the interview or withdraw from the study at any point.
- Please avoid sharing sensitive/personal information about specific patients.
- I will be recording the interview so it can be transcribed later. Everything you say will be kept confidential and the audio recording and transcription of what you say will be stored in a safe and secure place.
- Have you read the Participant Information Sheet?
- Do you have any questions before we begin?

Great, let get started are you happy if I start the audio recording. The recording is not capturing video

*[recording starts]*

**Participant Background information:**

- Firstly, I’m going to ask you a few general questions about yourself.
- How long have you been practicing podiatry?
- Where in Aotearoa New Zealand do you practice?
- What your typical case load looks like (i.e., general care vs MSK).

**Opening questions:**

1. How often do you come across foot osteoarthritis in your practice? And what joints do you commonly see that are affected by OA?
   - *Prompts: For example, how many cases would you come across* ***each week****, month or year, or what* ***proportion of your case load*** *would have foot osteoarthritis?*
   - *First MTPJ, MIDFOOT*
2. How often would these cases have been previously diagnosed with osteoarthritis prior to seeing you?
3. How would you define osteoarthritis? – What words come to mind? – Get them to elaborate
4. When you see patients with foot osteoarthritis is it typically their chief complaint or a secondary finding and/or complaint? *- Is it something the patient has just accepted as an inevitable part of aging?*

**THE NEXT PART OF THE INTERVIEW WILL INCLUDE KEY QUESTIONS RELATED TO DIAGNOSING FOOT OA**

1. What subjective and objective assessments do you use and how do you use them to reach your diagnosis foot osteoarthritis- these can be specific to individual joints?

- *Prompts: palpation, observations, subjective history, imaging, range of motion tests, strength testing, pressure analysis, gait analysis, outcome measures, pain scales…*
- *Are any joint specific?*

1. In your experience, of the assessments you have listed, is there any assessment(s) that you think is most helpful in diagnosing foot osteoarthritis? – *once more these can me specific to individual foot joints*
2. Are there any other conditions that you are trying to rule out when performing these assessments?

- *Prompts: Rheumatoid arthritis, turf toe, gout flare…*

1. Is there anything else you use, or refer for, to assist you with your diagnosis?

- *Prompts: specific subjective information from the patient (i.e., previous foot injuries, referral for imaging, activity limitations*
- ***If mentions imaging****- get them to elaborate on choice of modality*

**Prompt to discuss other foot joint*

**THE NEXT PART WILL INCLUDE KEY QUESTIONS RELATED TO TREATMENT AND MANAGEMENT OF FOOT OA**

1. In your experience, what does your short-term management include for a patient with foot osteoarthritis – Again, this can be joint specific

- *Prompts: what treatment interventions do you use? offloading? Taping? Orthotics? Braces? Splints? Footwear? Insole modifications (felt or materials)? Moonboot? Avoidance of certain activities? Activity modification? Education? Education on weight management? Non-steroidal anti-inflammatories (NSAIDs) oral/topical?*

1. In your experience, what does your long-term management include? -What treatment interventions do you use- *Do you follow any specific timeframes?*

- *Prompts: as above, plus surgical referral*

1. What influences your decision to opt for a specific treatment intervention?

- *Prompts: For example, what characteristics/qualities would qualify for you to make the decision to use orthotic therapy for one patient and an orthopaedic referral for another?*

1. In your experience, which treatment intervention have you had most success with when managing foot osteoarthritis? – *Joint specific (first MTPJ /Midfoot)*
2. Does your chosen intervention depend on their severity of foot osteoarthritis? If so, how do you determine degree of severity?
3. How often do you review your chosen treatments for patient with foot OA? – what indicators prompt you to modify your treatment intervention?

- *Prompts: Do you have any working theories on how this may help your patients?*

1. **If you use orthotic therapy, can you describe the modifications and materials you use?**

- *Prompts: forefoot modifications (Morton’s extension, cluffy extension, kinetic wedge, lateral FF, reverse Morton’s, plantar pad, ‘u’ cut out plantar pad, plantar pad, MT dome, MT bar), midfoot (arch fill), rearfoot (varus, valgus, heel rise, heel cushion) …*
- *Prompts: what shell materials…..*

1. **At what point would you refer for diagnostic imaging?- Repeat Imaging?**

• *Prompts: Initial assessment, after 3 months, 6 months, etc, of no improvement in symptoms*

1. **What imaging technique would you refer for and why?**

- *Prompts: Ultrasound/ X-ray*

1. **In your experience, at what point do you refer a patient to for more invasive/non-conservative treatment or diagnostic options?**

*• Prompts: Initial assessment, after 3 months, 6 months, etc, of no improvement in symptom, based on imaging results, or to obtain diagnostic imaging or treatments that are outside a NZ registered podiatrists’ scope of practice.*

**Prompt to discuss other foot joint*

**[CONCLUDING STATEMENT AND QUESTION]:**

Thank you all for your valuable input. This interview has been very insightful.

To finish off, I’d like to ask if you have any final comments on what you thought was the most important points from this interview, or if there is anything else you would like to add that we haven’t covered yet?

[recording stops]
