# Supplementary material for: Experiences of New Zealand Podiatrists Providing Podiatry Care to People With Foot Osteoarthritis
Source: J Foot Ankle Res. 2025 Dec 17;18(4):e70108. doi: 10.1002/jfa2.70108 (PMC12712349; doi:10.1002/jfa2.70108)
Supplement: Supplementary file 3 — Supporting Information S3 [file JFA2-18-e70108-s003.docx]

**Audit Trail Table: Experiences of NZ Podiatrists Providing Care for Foot Osteoarthritis**

| Raw Data (Verbatim Quote) | Participant | Initial Code | Coding Memo | Potential Theme | Theme Rationale | Final Theme |
| --- | --- | --- | --- | --- | --- | --- |
| “OA is more commonly presented as a secondary finding rather than the patient’s main concern.” | P8 | OA as incidental finding | Foot OA often not chief complaint; importance of identification | Chief complaint vs incidental finding | 8/10 participants noted OA was rarely the primary complaint | Assessment: Chief complaint vs incidental finding |
| “I would ask questions related to location of pain, severity of pain, which movements exacerbated pain …” | P8 | Subjective history: pain pattern | Pain/stiffness pattern critical for diagnosis | Obtaining patient history through subjective interview | Reported by all participants | Assessment: Subjective history |
| “Pain on the dorsal aspect of the joint, combined with restriction of first MTP joint dorsiflexion…” | P1 | Targeted objective assessments (MTPJ ROM, palpation) | Use of performance/impairment measures | Foot OA targeted objective assessments | Listed suite of tests used consistently | Assessment: Targeted objective assessments |
| “I would assess to see if there are any modifiable biomechanical factors that could be addressed…” | P8 | Identify modifiable biomechanical factors | Individualised gait/kinematics considerations | Individual biomechanical factors | Recurs in the majority of interviews | Assessment: Individual biomechanical factors |
| “There’re probably two reasons why I would refer for X-ray…” | P10 | Imaging as confirmatory/management guidance | Plain radiography most used; timing varies | Further investigations | Reported by nine participants | Assessment: Further investigations |
| “Wear and tear arthritis’ and ‘joint space narrowing’ are the simple words that I would use to explain OA to patients” | P2 | Use of impairment-based language | Outdated terminology; need for contemporary narrative | Knowledge and language | Emphasis on education; outdated terms noted | Management: Knowledge and language |
| “Different treatments work for different people… trial-and-error approach” | P8 | Iterative management due to uncertainty | Facilitate vs restrict joint motion dilemma | Clinical uncertainty necessitates an iterative approach | Consensus to facilitate movement until it is too painful | Management: Iterative approach |
| “Education on footwear is fundamental; we don’t do anything until we have the footwear right” | P3 | Footwear-first strategy | Rocker-sole for MTPJ OA; stability for midfoot OA | Podiatric therapeutic approaches (Footwear) | Common recommendation across participants | Management: Podiatric therapeutic approaches |
| “I will often apply a strapping technique to immobilise and resist dorsiflexion of the first MTPJ…” | P8 | Strapping for symptom management | Strapping as diagnostic and therapeutic tool | Podiatric therapeutic approaches (Strapping) | Used to guide orthotic therapy | Management: Podiatric therapeutic approaches |
| “I tend to gravitate towards customs over prefabs for chronic conditions such as OA…” | P3 | Custom orthoses for chronic OA | Customisation for durability and flexibility | Podiatric therapeutic approaches (Orthoses) | Chronic nature justifies custom devices | Management: Podiatric therapeutic approaches |
| “I will often incorporate manual therapy, joint mobilisation, stretching…” | P3 | Exercise prescription | Reduce proximal compensations, improve function | Podiatric therapeutic approaches (Exercise) | Often recommended alongside other interventions | Management: Podiatric therapeutic approaches |
| “So pharmaceutically… I would recommend consulting a pharmacist or GP” | P3 | Interdisciplinary referral pathways | Escalation to specialist if conservative care fails | Referral pathways to other health professionals | Reported by eight participants | Management: Referral pathways |
| “A lot of my patients don’t have the privilege of purchasing new shoes and orthoses…” | P6 | Cost & access constraints | Health inequities influence choices | Management influences | Cost/region access shaping practice | Management: Management influences |
| “How active do they want to be? … Some patients come in and just want to do their day-to-day activity without pain…” | P1 | Patient goals influence management | Individual goals shape intervention choices | Management influences | Patient-centred management | Management: Management influences |
